# In Vitro Methylene Blue and Carboplatin Combination Triggers Ovarian Cancer Cells Death

**DOI:** 10.3390/ijms252011005

**Published:** 2024-10-13

**Authors:** Jorgelindo da Veiga Moreira, Laurent Schwartz, Mario Jolicoeur

**Affiliations:** 1 Research Laboratory in Applied Metabolic Engineering, Department of Chemical Engineering, Polytechnique Montréal, Centre-Ville Station, P.O. Box 6079, Montréal, QC H3C 3A7, Canada; jorgelindo.daveiga@polymtl.ca; 2 Assistance Publique des Hôpitaux de Paris, Avenue Victoria, 75003 Paris, France; dr.laurentschwartz@gmail.com

**Keywords:** ovarian cancer, cancer metabolism, methylene blue, Warburg effect, mitochondria

## Abstract

Ovarian cancer presents a dire prognosis and high mortality rates, necessitating the exploration of alternative therapeutic avenues, particularly in the face of platinum-based chemotherapy resistance. Conventional treatments often overlook the metabolic implications of cancer, but recent research has highlighted the pivotal role of mitochondria in cancer pathogenesis and drug resistance. This study delves into the metabolic landscape of ovarian cancer treatment, focusing on modulating mitochondrial activity using methylene blue (MB). Investigating two epithelial ovarian cancer (EOC) cell lines, OV1369-R2 and OV1946, exhibiting disparate responses to carboplatin, we sought to identify metabolic nodes, especially those linked to mitochondrial dysfunction, contributing to chemo-resistance. Utilizing ARPE-19, a normal retinal epithelial cell line, as a control model, our study reveals MB’s distinct cellular uptake, with ARPE-19 absorbing 5 to 7 times more MB than OV1946 and OV1369-R2. Treatment with 50 µM MB (MB-50) effectively curtailed the proliferation of both ovarian cancer cell lines. Furthermore, MB-50 exhibited the ability to quell glutaminolysis and the Warburg effect in cancer cell cultures. Regarding mitochondrial energetics, MB-50 spurred oxygen consumption, disrupted glycolytic pathways, and induced ATP depletion in the chemo-sensitive OV1946 cell line. These findings highlight the potential of long-term MB exposure as a strategy to improve the chemotherapeutic response in ovarian cancer cells. The ability of MB to stimulate oxygen consumption and enhance mitochondrial activity positions it as a promising candidate for ovarian cancer therapy, shedding light on the metabolic pressures exerted on mitochondria and their modulation by MB, thus contributing to a deeper understanding of mitochondrial dysregulation and the metabolic underpinnings of cancer cell proliferation.

## 1. Introduction

Ovarian cancer presents a significant challenge in oncology, boasting the highest fatality rate among gynecological cancers [1]. A staggering 70% of epithelial ovarian cancer (EOC) cases are diagnosed at an advanced stage, with most proving unresponsive to current platinum-based chemotherapy [2]. Despite initial responses in up to 80% of patients, drug resistance inevitably develops, leading to relapse and mortality [3]. Standard treatments for platinum-resistant ovarian cancer, such as pegylated liposomal doxorubicin, paclitaxel, and topotecan, offer limited efficacy [2,4]. To address this, various pre-clinical models, including 2D, 3D, and ex vivo cultures of EOC, have been proposed to assess carboplatin sensitivity [5,6]. From a metabolic perspective, chemo-resistance in ovarian cancer is associated with the following two key phenomena: the Warburg effect, characterized by enhanced aerobic glycolysis, and heightened glutaminolysis [7,8]. While these metabolic features are common in cancer cells, they are particularly pronounced in chemo-resistant ovarian cancer cells [9,10]. Over the past decade, research has extensively explored cancer cell metabolism, focusing on mitochondrial involvement, as proposed by Otto Warburg, who described cancer as a metabolic disease driven by mitochondrial dysfunction [11,12,13], leading to increased anabolism and decreased catabolism [14,15,16,17,18,19]. Methylene blue (MB), a long-established antibacterial agent, was employed to stimulate mitochondrial electron transport chain and oxidative phosphorylation (OXPHOS) activity, resulting in decreased lactate production [20,21,22,23]. Recent studies on Chinese hamster ovary (CHO) cells have further emphasized the pivotal role of mitochondria in carbon flux regulation [24]. Building upon these insights, our study explores the potential of MB as a cancer treatment candidate, aiming to disrupt cancer cell reprogramming reliant on glycolysis by enhancing mitochondrial activity. We selected two EOC lines, OV1369-R2 and OV1946, with differing responses to carboplatin [6,25], with OV1369-R2 demonstrating resistance and OV1946 displaying intermediate sensitivity. Utilizing ARPE-19 as a normal retinal epithelial control model, our findings demonstrate MB’s efficacy in inducing ovarian cancer cells, especially when combined with cisplatin. These results underscore mitochondrial energetics as a critical vulnerability in cancer cells and suggest that “long-term” in vitro exposure (48 h-to-72 h) to methylene blue may overcome chemo-resistance in ovarian cancer cells.

## 2. Results

### 2.1. Methylene Blue Cellular Intake Is Associated with Lower ATP Amount in Ovarian Cancer Cells

**Figure 1:**
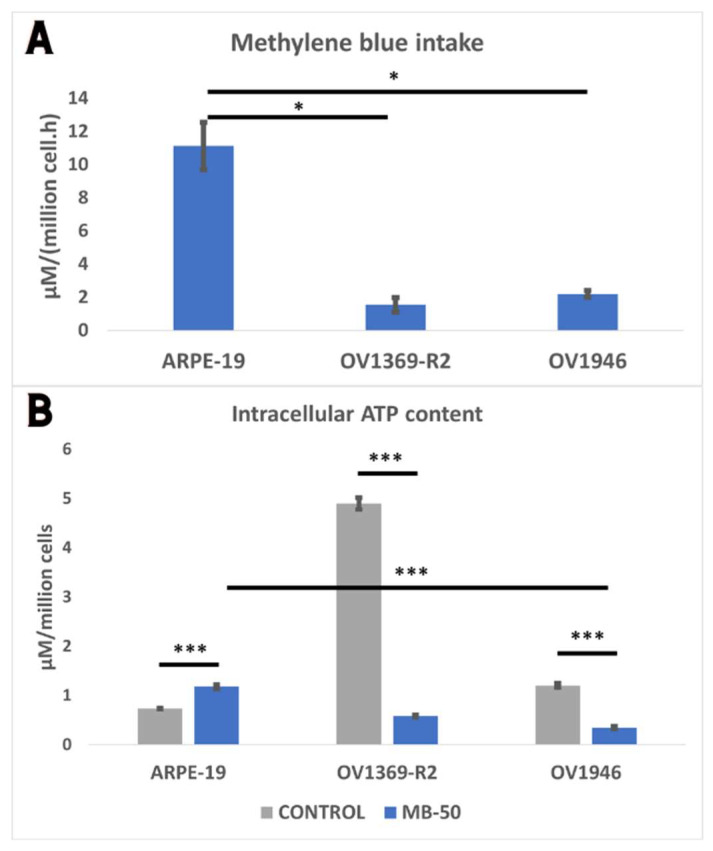
(**A**) Differential intake of methylene blue (MB) by ARPE-19, OV1369-R2, and OV1946 cell lines. ARPE-19 exhibited higher MB intake compared to the cancerous lines, with approximately 5-fold and 7-fold greater accumulation than OV1369-R2 and OV1946, respectively. (**B**) Modulation of free ATP levels in response to MB treatment. ARPE-19 cells showed an increase in ATP content upon MB treatment, while both OV1369-R2 and OV1946 displayed an MB-inhibitory effect on ATP levels. The adjusted *p*-value ≤ 0.05 were considered significant and the notations of * (*p* ≤ 0.05), and *** (*p* ≤ 0.001) were used.

Assessment of MB uptake revealed significantly higher absorption by the normal control cell line ARPE-19 compared to the cancerous cell lines OV1369-R2 and OV1946 (Figure 1A). Normalized to cell number, ARPE-19 accumulated MB at a rate of 11.13 ± 1.43 µM/(million cells·h), approximately 5 to 7 times greater than OV1369-R2 (1.55 ± 0.46 µM/(million cells·h)) and OV1946 (2.20 ± 0.21 µM/(million cells·h)) (*p* ≤ 0.05). The reduced permeability of cancer cell membranes, including mitochondria, suggests limited internalization of MB into their cytoplasm or mitochondria, consistent with their preference for aerobic glycolysis, a hallmark of the Warburg effect. Further investigation into MB’s impact on cell energetics focused on total ATP content after 24 h MB-50 incubation. The results revealed a significant inhibitory effect of MB on cancer cell ATP levels (Figure 1B). Under normal growth conditions, OV1369-R2 and OV1946 exhibited higher intracellular ATP content per cell compared to controls, with values of 4.90 ± 0.12 and 1.21 ± 0.04 µM/million cells, respectively (*p* ≤ 0.001). However, they displayed greater sensitivities to MB-50 treatment, resulting in a remarkable 88% and 71% decrease in total ATP levels, respectively. In contrast, ARPE-19 responded differently to MB-50, exhibiting a 60% increase in ATP content, rising from 0.74 ± 0.01 µM/million cells to 1.18 ± 0.04 µM/million cells upon treatment (*p* ≤ 0.001). This inverse relationship between MB’s inhibitory effect on cancer cell energy demand and mitochondrial dye absorption prompts further exploration into its potential inhibitory effects on cancer cell proliferation.

### 2.2. Cancer Cells Proliferation Is Sensitive to Methylene Blue

**Figure 2:**
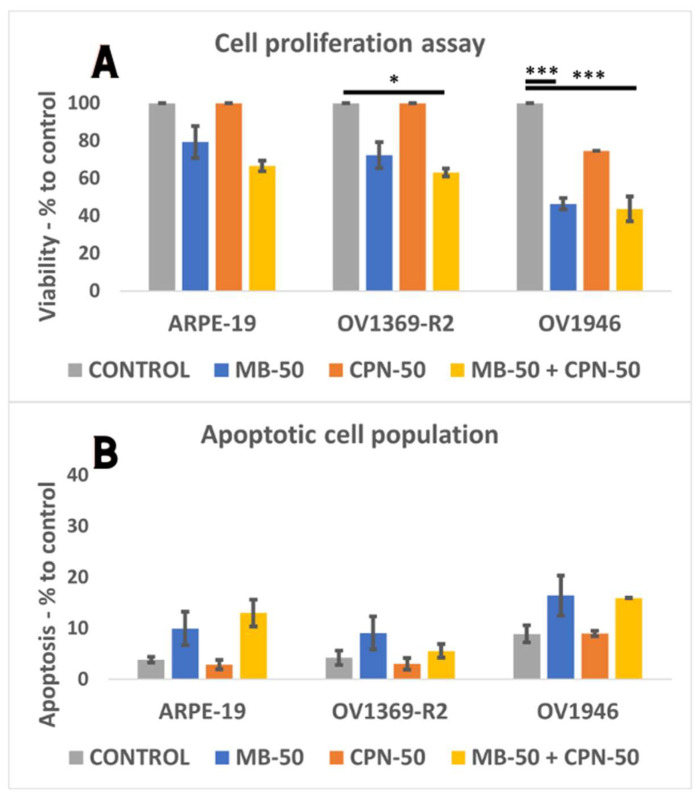
Sensitivity of cancer cells to methylene blue and carboplatin treatment. (**A**) Cells were incubated for 24 h under different conditions: 50 µM methylene blue (MB-50), 50 µM carboplatin (CPN-50), and the combination of MB-50 and CPN-50. The “CONTROL” condition included the solvent of MB and CPN, all in OSE culture medium. The combination of MB-50 and CPN-50 did not show a significant effect compared to MB-50 alone. Notably, OV1946 showed sensitivity to CPN-50, whereas ARPE-19 and OV1369-R2, known to be resistant to carboplatin treatment, did not. (**B**) Induction of apoptosis was observed to a limited extent under 24 h treatment conditions for all three cell lines. The apoptotic cell population was slightly higher in the intermediate chemo-sensitive cell line OV1946. The adjusted *p*-value ≤ 0.05 were considered significant and the notations of * (*p* ≤ 0.05), and *** (*p* ≤ 0.001) were used.

The impact of different treatment conditions on proliferation and apoptosis induction was assessed in the normal cell line ARPE-19 and the cancerous cell lines OV1369-R2 and OV1946 following overnight culture and subsequent 24 h incubation. Conditions included vehicle control (OSE culture medium), MB-50, CPN-50, and the combination of MB-50 and CPN-50 (IC_50_s provided in Figure S1). Flow cytometry by fluorescence-activated cell sorting (FACS) analysis was performed to evaluate treatment effects on cell proliferation and apoptosis. As shown in Figure 2A, neither ARPE-19 nor OV1369-R2 exhibited significant sensitivity to CPN-50 and MB-50, confirming OV1369-R2′s known chemo-resistance and OV1946′s intermediate sensitivity. However, both cancer cell lines showed significant sensitivity to MB-50 when combined with CPN-50 (*p* ≤ 0.05 and *p* ≤ 0.001 for OV1369-R2 and OV1946, respectively). OV1946 demonstrated the highest sensitivity to MB-50 (*p* ≤ 0.001), resulting in only 46 ± 3% proliferation compared to 72 ± 7% for OV1369-R2 (*p* ≤ 0.01) and 79 ± 9% for ARPE-19 after 24 h of treatment. The combination of MB-50 and CPN-50 did not significantly increase MB-50′s effect on cancer cell proliferation. Regarding apoptosis induction, MB-50 exhibited a modest effect on all three lines that was not significantly different from the control conditions (Figure 2B). However, OV1946 displayed slightly higher, but not significant apoptosis levels, particularly under MB-50 conditions, whether combined with CPN-50 or not. The adjusted *p*-value ≤ 0.05 were considered significant and the notations of * (*p* ≤ 0.05), ** (*p* ≤ 0.01), and *** (*p* ≤ 0.001) were used.

### 2.3. Warburg Effect and Glutaminolysis Are Inhibited in Chemo-Sensitive Cell Line

**Figure 3:**
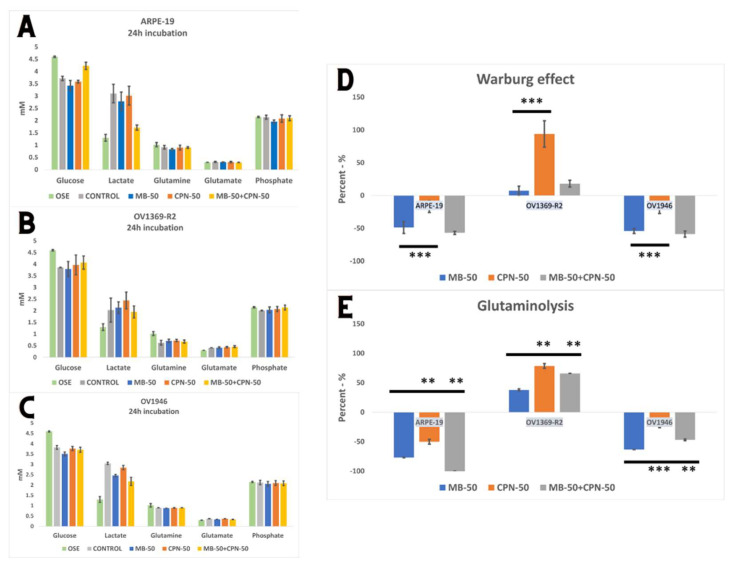
Glutaminolysis and Warburg effect in ovarian cancer cells. (**A**–**C**) The glycolytic metabolic profile of cells was assessed under various treatment conditions, and the concentrations of glucose, lactate, glutamine, glutamate, and phosphate in the culture medium were measured after a 24 h treatment period. (**D**,**E**) Methylene blue treatment exerts inhibitory effects on both the Warburg effect and glutaminolysis in ARPE-19 and the chemo-sensitive OV1946 cell line. Specifically, MB significantly reduces the Warburg effect, as indicated by decreased lactate production and glucose consumption. Additionally, MB suppresses glutaminolysis, leading to reduced glutamate production and glutamine consumption. These metabolic alterations are consistent with the observed inhibition of cell proliferation. In contrast, the chemo-resistant OV1369-R2 cell line exhibited an opposing response to MB treatment. Here, MB slightly enhanced the Warburg effect and upregulated glutaminolysis. Notably, the combination of MB with carboplatin (CPN-50) did not significantly augment the Warburg effect. The opposite effect was observed on glutaminolysis, with a significant effect of drug combination (*p* ≤ 0.01). The adjusted *p*-value ≤ 0.05 were considered significant and the notations of ** (*p* ≤ 0.01), and *** (*p* ≤ 0.001) were used.

Understanding methylene blue’s metabolic effects inhibiting cancer cell proliferation was a pivotal aim of this study. We examined variation in five extracellular metabolites critical for cancer cell metabolism over a 24 h culture period (Figure 3A–C). Glucose and glutamine, essential substrates in central carbon metabolism, provide carbon resources for biomass synthesis. Proliferating cells exhibit high lactate production through aerobic glycolysis/the Warburg effect and glutamate production via glutaminolysis, well-documented cancer cell characteristics. To assess methylene blue and carboplatin impact, we defined the Warburg effect (WE) as net lactate (LAC) production/net glucose (GLC) consumption (∆LAC/∆GLC) and glutaminolysis (∆GLU/∆GLN) as net extracellular glutamate (GLU) production/net glutamine (GLN) consumption at 24 h post-treatment. ARPE-19 and OV1946 ovarian cancer line exhibited similar WE and glutaminolysis modulation under the proliferation conditions (Figure 3D,E). Under MB-50, WE was strongly inhibited in ARPE-19 (−49 ± 9%) and OV1946 (−54 ± 2%), less under CPN-50 alone, −17 ± 7% and −18 ± 5%, respectively (*p* ≤ 0.001). The combination condition had no significant additional effect on these two cell lines compared with MB-50 alone. However, OV1369-R2 showed slight upregulation under MB-50 alone and in combination with CPN-50 but increased by 94% ± 20% under CPN-50 alone. Glutaminolysis was significantly inhibited under MB-50 in ARPE-19 (−77 ± 1%) and OV1946 (−63 ± 1%) but upregulated in OV1369-R2 (+38 ± 4%). This distinctive OV1369-R2 behavior underscores central carbon metabolism’s importance in cancer cell treatment response. Glutaminolysis was more upregulated under CPN-50 than MB-50 in OV1369-R2 (*p* ≤ 0.01), attenuated when combined with MB-50. This metabolic uncoupling post-carboplatin or methylene blue treatments suggests mitochondrial metabolism’s role in drug resistance or intrinsic cancer cell’s adaptive characteristics. Additionally, metabolic reprogramming, especially sustaining aerobic glycolysis and glutaminolysis, is a hallmark of cancer cells, particularly cancer stem cells (CSCs).

### 2.4. Long-Term Exposure to Methylene Blue Induces Increased Apoptosis in Ovarian Cancer Cells

**Figure 4:**
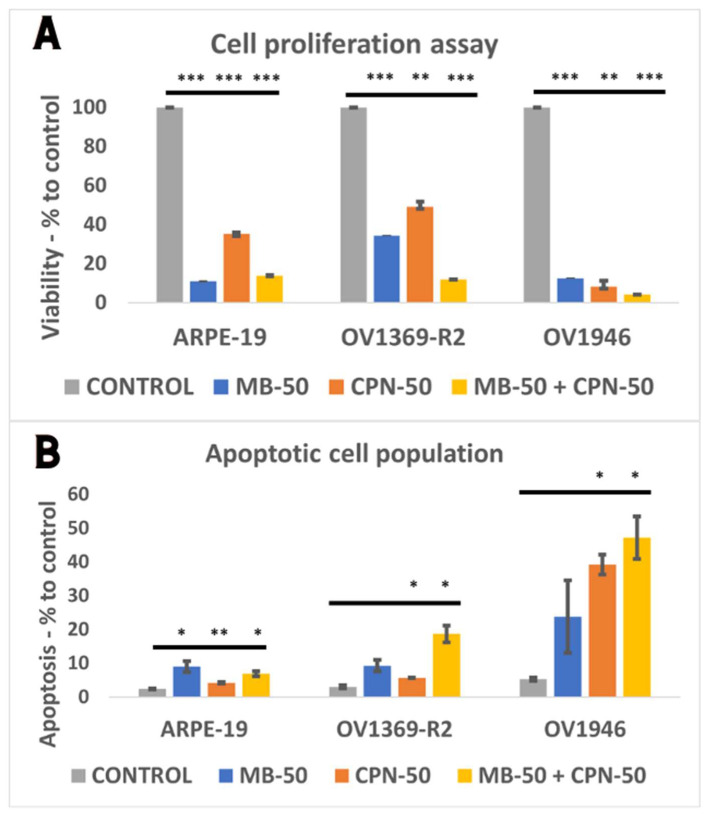
Methylene blue and carboplatin combination trigger the apoptotic pathway in ovarian cancer cells. Cells were treated for 24 h with 50 µM methylene blue (MB-50), 50 µM carboplatin (CPN-50), or a combination of MB-50 and CPN-50. After treatment, cells were rinsed and incubated for an additional 24 h in normal OSE culture medium. The “CONTROL” condition contained the solvents used for methylene blue and carboplatin in OSE culture medium. This illustrates the impact of these treatments on cell viability (**A**) and apoptotic cell populations (**B**), demonstrating that the combination of MB-50 and CPN-50 significantly reduces cancer cell viability (**A**) and increases apoptosis (**B**) compared to individual treatments and controls. The adjusted *p*-value ≤ 0.05 were considered significant and the notations of * (*p* ≤ 0.05), ** (*p* ≤ 0.01), and *** (*p* ≤ 0.001) were used.

After a 24 h proliferation assay with 50 µM methylene blue (MB-50), the OV1369-R2 and OV1946 ovarian cancer cell lines exhibited sensitivity to methylene blue, with no significant effect when combined with 50 µM carboplatin (MB-50 + CPN-50) (Figure 2). To further investigate the potential of MB-50 in inducing apoptosis in OV1369-R2 cells, an initial 24 h treatment was followed by rinsing and re-incubation in normal OSE culture medium for an additional 24 h, totaling 48 h of “long-term” incubation. Flow cytometry (FACS) analysis demonstrated a reduced replicative capacity in all three cell lines, even after resuming normal growth conditions (Figure 4A). Both cancer cell lines showed significantly reduced viability, especially under MB-50 + CPN-50 combination (*p* ≤ 0.001). Notably, OV1946 remained highly sensitive, with less than 4% ± 0.1 of cells remaining compared to control (*p* ≤ 0.001). This decrease in viability was associated with a significant increase in the apoptotic cell population in both OV1369-R2 and OV1946 cells under MB-50 + CPN-50 combination condition (Figure 4B) (*p* ≤ 0.05). Increased apoptosis was reported in OV1946 cells, resulting in a 47 ± 6% apoptotic population (*p* ≤ 0.05), compared to 19 ± 3% in OV1369-R2 and 7 ± 1% in ARPE-19 cells (Figure 4B).

### 2.5. Methylene Blue Induces a Significant Increase in Oxygen Consumption Rate (OCR) in Cancer Cell Cultures

**Figure 5:**
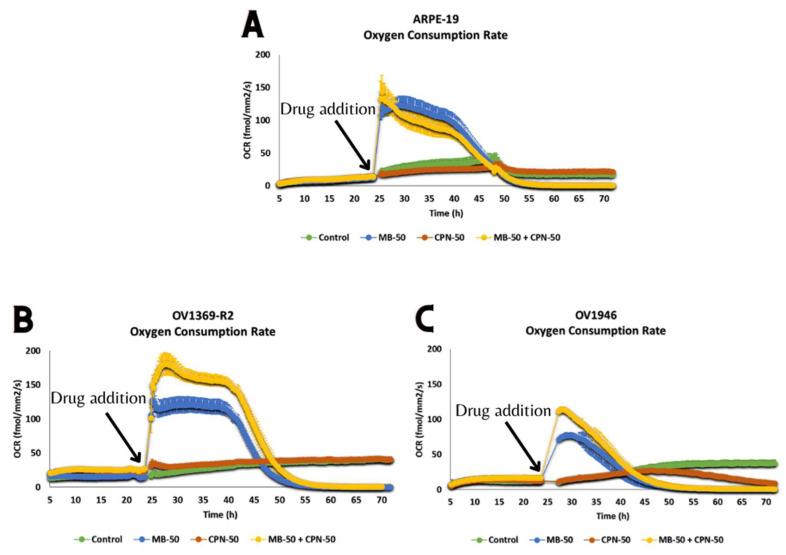
Oxygen consumption rate (OCR) in ARPE-19, OV1369-R2, and OV1946 cell lines. (**A**) ARPE-19 cells showed a marked increase in OCR upon treatment with 50 µM methylene blue (MB-50) and further enhancement when combined with 50 µM carboplatin (CPN-50). (**B**) OV1369-R2 cells exhibited a significant rise in OCR following MB-50 treatment, with an even greater increase when combined with CPN-50, indicating enhanced sensitivity to the combined treatment. (**C**) In OV1946 cells, MB-50 treatment resulted in a moderate increase in OCR compared to OV1369-R2 cells. However, the combination of MB-50 and CPN-50 led to a higher OCR than MB-50 alone.

To elucidate the effects of methylene blue (MB) and its combination with carboplatin (CPN) on cancer cell respiration, we monitored the oxygen consumption rate (OCR) over three days under standard growth conditions (5% CO_2_, 37 °C) and various drug treatments (MB-50, CPN-50, MB-50 + CPN-50). The addition of MB at 24 h prompted a rapid and significant response across all three cell lines—ARPE-19, OV1369-R2, and OV1946—evidenced by a marked increase in OCR. In particular, OV1369-R2 displayed a notable rise from a basal cell respiration of 16 ± 3 fmol/mm^2^/s prior to MB addition to a peak of 128 ± 10 fmol/mm^2^/s. Subsequently, OCR stabilized for 20 h under MB-50 treatment before declining below detection limits, likely due to metabolic pressure exerted by MB on the mitochondrial respiratory pathway or oxidative phosphorylation (OxPhos) energetics. Conversely, CPN-50 exhibited no discernible effects on OCR in ARPE-19 and OV1369-R2 cell lines (Figure 5A,B). However, the combination of MB-50 and CPN-50 notably increased OCR in the OV1369-R2 cell line. Similar trends were observed in the OV1946 cell line, albeit with a less pronounced OCR response compared to OV1369-R2 or ARPE-19 (Figure 5A–C). Notably, OV1946 demonstrated sensitivity to CPN, with OCR reduction below detection limits starting from 45 h onwards. Notably, the OCR values for all three cell lines treated with methylene blue, with or without carboplatin, declined below initial OCR levels. While this decline may partly stem from cell death and induced apoptosis, it does not entirely account for the observed dynamics, as the rapid decrease in OCR observed 20 h after MB addition surpassed the decline in cell viability.

## 3. Discussion

Previous investigations conducted by our research team have elucidated the crucial involvement of mitochondria in determining the allocation of carbon resources between energy deprivation (catabolism) and the synthesis of biomass building blocks (anabolism). To replicate the inflammatory microenvironment typical of cancer [26,27], we subjected Chinese hamster ovary (CHO) cells to osmotic shocks in culture [28]. These shocks induced an acceleration of the Warburg effect in CHO cells and a notable decrease in mitochondrial capacity (h = ∆p/∆Ψm). These metabolic changes align with the characteristic signatures described by Otto Warburg in cancer cells [7]. In our present study, our findings corroborate a pronounced aerobic glycolysis/Warburg effect in the OV1369-R2 and OV1946 cancer cell lines, with OV1946 exhibiting a particularly robust effect (Figure 3C). Additionally, both cancer cell lines demonstrated elevated rates of glutaminolysis compared to the normal epithelial cell line ARPE-19. This heightened glutaminolysis reflects the increased demand for nitrogen resources by cancer cells for nucleotide and protein synthesis. Mitochondria utilize ammonium and glutamate derived from glutaminolysis for the conversion of Krebs cycle intermediates and biomass synthesis. Consequently, this metabolic imbalance tipped towards anabolism in cancer cells leads to a heightened energetic demand and an abundance of free cellular ATP (Figure 1B). A strategy to redirect cellular energetics in cancer cells and promote mitochondrial catabolism involves the use of methylene blue (MB). As a potent redox agent, MB remains unmetabolized by cells and is primarily consumed by mitochondria. Its notable role lies in serving as an alternative electron transport chain (ETC). Our results demonstrate that MB induces enhanced cell respiration capacity, even in cancer cell lines, challenging the prevailing notion of impaired mitochondrial activity in cancer cells (Figure 5B,C) [29]. Disrupting the cellular energy balance through MB-50 treatment leads to a significant reduction in total cellular ATP levels in both cancer cell lines, OV1369-R2 and OV1946, while total ATP is enhanced in the normal cell line ARPE-19 (Figure 1B). This suggests that ARPE-19 cells can increase mitochondrial ATP synthesis in response to the high flux of mitochondrial MB (Figure 1A,B), in contrast to the cancer cells. Furthermore, our results reveal a direct correlation between the rate of MB uptake by the cells and the inhibition of the Warburg effect and glutaminolysis. Both metabolic phenomena are notably suppressed in ARPE-19 and OV1946, the two cell lines exhibiting higher MB consumption (Figure 3A,C–E). Conversely, the opposite trend is observed in OV1369-R2, the chemo-resistant cell line. Interestingly, the effect of MB on chemo-resistant cancer cells bears resemblance to microgravity experiments conducted on lymphoblastic cancer cell cultures [30]. In that study, researchers demonstrated the anticancer effect of time-averaged simulated microgravity (taSMG) on human Hodgkin’s lymphoma (HL) cells in culture. Consistent with our findings, they reported a decrease in intracellular ATP levels, which, in turn, led to increased generation of reactive oxygen species (ROS) and pronounced autophagy in HL cells. Subsequently, we assessed apoptosis induction in cell populations after short-term (24 h) and long-term (48 h) treatments with CPN-50, MB-50, or their combination (MB-50 + CPN-50). The results demonstrated a higher apoptotic response in the cancer cell lines under the MB-50 + CPN-50 condition (Figure 4B). Considering that apoptosis is an energetically demanding process for cells, coupled with the decrease in ATP and lactate production from glycolysis (Figure 3A–C), the observed reduction in long-term cell proliferation under MB-50 + CPN-50 treatment is rationalized (Figure 4A). The intensified apoptosis in cancerous cell lines may be attributed to an energy deficiency that fails to meet the anabolic demands of these cells. This deficiency is particularly pronounced in OV1946 cells, which display heightened sensitivity to CPN-50 and MB-50. Moreover, the augmented apoptosis in OV1369-R2 cells under the likely “synergistic effect” of MB-50 and CPN-50 sheds light on the pivotal role of mitochondria in chemo-resistance (Figure 4B). The concurrent elevation of the Warburg effect and glutaminolysis following CPN-50 treatment in OV1369-R2 represents critical markers of chemo-resistance and mitochondrial function. This response to CPN-50 treatment underscores one of the cancer cell’s vulnerabilities: the regulation of mitochondrial redox potential. Consequently, the development of photodynamic therapies utilizing MB in combination with mitochondrial singlet oxygen production has gained momentum [15,31,32,33].

## 4. Materials and Methods

### 4.1. Statistical Analysis

Data are presented as mean ± the standard error of the mean (*n* = 3). Statistical analysis was performed with Python 3.8 and Excel 2409. Values of adjusted *p*-value ≤ 0.05 were considered significant and the notations of * (*p* ≤ 0.05), ** (*p* ≤ 0.01), and *** (*p* ≤ 0.001) were used for the comparison versus the control group by ANOVA followed by Tukey HSD post hoc tests.

### 4.2. Cell Lines and Culture Medium

Two epithelial ovarian cancer cell lines, OV1369-R2 and OV1946, derived from patient ascites and sourced from the OvCAN collection, were utilized in this study with the explicit permission of the CHUM (Centre Hospitalier de l’Université de Montréal) Research Ethics Board (BD 04.002). The normal retinal epithelial cell line, ARPE-19, was procured from ATCC (CRL-2302) and employed as a suitable control model. OV1369-R2 and OV1946 exhibited resistance and an intermediate response, respectively, to carboplatin treatment in 2D cultures. The three cell lines were maintained in complete OSE medium (Wisent, Cat. 316-030-CL, Saint-Jean-Baptiste, QC, Canada) with the addition of 10% fetal bovine serum (Wisent, Cat. 080-150, Saint-Jean-Baptiste, QC, Canada), 250 µg/mL Amphotericin B (Wisent, Cat. 450-105-QL, Saint-Jean-Baptiste, QC, Canada), and 50 mg/mL Gentamicin (Wisent, Cat. 450-135-XL, Saint-Jean-Baptiste, QC, Canada). Before experiments were initiated, the cells were incubated at 37 °C in an environment controlled to 21% O_2_ and 5% CO_2_.

### 4.3. Methylene Blue and Carboplatin Treatments

Various methylene blue (MB) concentrations, from 0 to 50 μM, were tested on the three cell lines to observe their responses within a 24 h period (see Supplementary Information). Among these, the 50 μM MB dose (MB-50) resulted in a 50% reduction in viability in OV1946, the most responsive cell line, leading us to choose this concentration for subsequent experiments. For the carboplatin assays (Sigma, Cat. C2538, Markham, ON, Canada), a consistent dose of 50 μM (CPN-50) was selected to allow direct comparison with the MB-50 effects. Notably, CPN-50 significantly exceeds the respective IC_50_ values of carboplatin for each cell line, namely OV1369-R2 (9.8 ± 1.0 μM) and OV1946 (3.4 ± 0.18 μM). Briefly, to ensure optimal adhesion to plates, cells were incubated for 16 h under normal growth conditions prior to initiating the experiments. Subsequently, the growth medium was replaced with MB-50, CPN-50, or the combination of MB-50 and CPN-50. Cells were further incubated for 24 h before conducting viability, apoptosis, and metabolomic assays.

### 4.4. Flow Cytometry Analysis

Cell viability was first assessed using the MTT assay kit (Sigma, Cat. 11465007001, Markham, ON, Canada), followed by flow cytometry analysis (BD Biosciences, Mississauga, ON, Canada) to reduce the potential for colorimetric interference from methylene blue (Laboratoire Chimie-plus, Saint-Paul-de-Varax, France). Viability was quantified by normalizing event counts to Precision Count Beads™ (BioLegend, Cat. 424902, Cedarlane, Burlington, ON, Canada) in the cell suspension. Apoptotic cell populations were similarly evaluated using Apotracker™ Green (BioLegend, Cat. 427402, QC, Canada) and normalized to the total cell count. Flow cytometry staining involved incubating 300 nM Apotracker™ Green per million cells in a 100 µL PBS solution. Detection was performed using the Alexa Fluor^®^ 488 channel (BD Biosciences, Mississauga, ON, Canada), and all flow cytometry data were analyzed with FlowJo™ v10.10 software (BD Biosciences, Mississauga, ON, Canada).

### 4.5. ATP Essay

The quantitative measurement of free cellular ATP levels was performed using the ATP Bioluminescent Assay Kit (Sigma, Cat. FLAA, Markham, ON, Canada). The assay involved the addition of luciferin and luciferase assay cocktail to the lysed cell extract, and the resulting light emission was directly proportional to the intracellular ATP concentration. The determination of free ATP levels in each growth condition, as well as following methylene blue treatment, was accomplished by referencing an ATP standard curve.

### 4.6. Oxygen Consumption Essay

The oxygen consumption rates were continuously monitored in the three cell lines under various conditions using the Resipher device (Lucid Scientist, Atlanta, GA, USA). The oxygen sensor was placed in 100 µL of cell culture within specially adapted Falcon 96-well plates (Corning, Cat. 353072, Corning, NY, USA). Measurements of oxygen consumption rate (OCR) began after 5 h, once the oxygen levels in the medium had reached surface saturation. These OCR readings reflected the dynamic oxygen exchange between the medium and cellular activity. To assess the impact of different drugs on cell respiration, they were introduced after 24 h of incubation, enabling the evaluation of their effects on oxygen consumption.

### 4.7. Metabolites Measurement

Glucose, glutamine, and phosphate consumption, as well as lactate and glutamate production, were evaluated by measuring their concentrations in the cell culture media before (OSE medium) and after treatment. Culture media were collected 24 h after treatments, and metabolite concentrations were determined using the Roche Cedex^®^ Bio Analyzer (REF: 06395554001, Laval, QC, Canada). All necessary calibrators, including glucose (REF: 06343732001, Laval, QC, Canada), glutamine (REF: 07395655001, Laval, QC, Canada), glutamate (REF: 07395582001, Laval, QC, Canada), phosphate (REF: 06990070001, Laval, QC, Canada), and lactate (REF: 06343759001, Laval, QC, Canada), were obtained from Roche and used according to the manufacturer’s protocols.

## 5. Conclusions

Ovarian cancer presents significant challenges due to its high mortality rates and limited response to standard therapies. Our investigation highlights the crucial role of mitochondrial metabolism in the development of chemo-resistance to platinum-based chemotherapy. The increased activity of glutaminolysis and the Warburg effect in chemo-resistant cells underscores the need to target mitochondrial energetics for effective ovarian cancer treatment. Methylene blue, acting as a mitochondrial stimulant, exhibits remarkable efficacy in inhibiting cancer cell proliferation by modulating central carbon metabolism. Particularly noteworthy is its potent effect in the carboplatin-resistant OV1369-R2 cell line, offering a promising therapeutic avenue for platinum-resistant ovarian cancer. Furthermore, methylene blue augments cancer cell respiration and respiratory capacity, revitalizing mitochondrial energetics vital for both cell survival and apoptosis. Our findings add to the mounting evidence highlighting the pivotal role of metabolic alterations affecting mitochondria in cancer development and may present a potential new avenue to mitigate chemotherapy resistance. By alleviating this metabolic pressure and enhancing mitochondrial function, methylene blue unveils novel prospects for innovative ovarian cancer treatments. In summary, our study provides valuable insights into the metabolic mechanisms driving the enhanced replicative potential of cancer cells and the dysregulation of mitochondrial energetics. It underscores the potential of methylene blue as an adjunctive therapeutic strategy against ovarian cancer and advocates for further in vitro and in vivo studies to better understand the molecular mechanisms underlying MB-induced ovarian cancer cells death.

## Data Availability

The data underlying this study are not publicly available currently. The authors are currently assessing the possibility of patenting the findings and protocols presented in the manuscript.

## Supplementary Materials

The following supporting information can be downloaded at: https://www.mdpi.com/article/10.3390/ijms252011005/s1.
