# Supplementary material for: In Vitro Methylene Blue and Carboplatin Combination Triggers Ovarian Cancer Cells Death
Source: Int J Mol Sci. 2024 Oct 13;25(20):11005. doi: 10.3390/ijms252011005 (PMC11507203; doi:10.3390/ijms252011005)
Supplement: Supplementary file 1 [file ijms-25-11005-s001.zip › ijms-3227951-supplementary.pdf]

Supplementary Materials

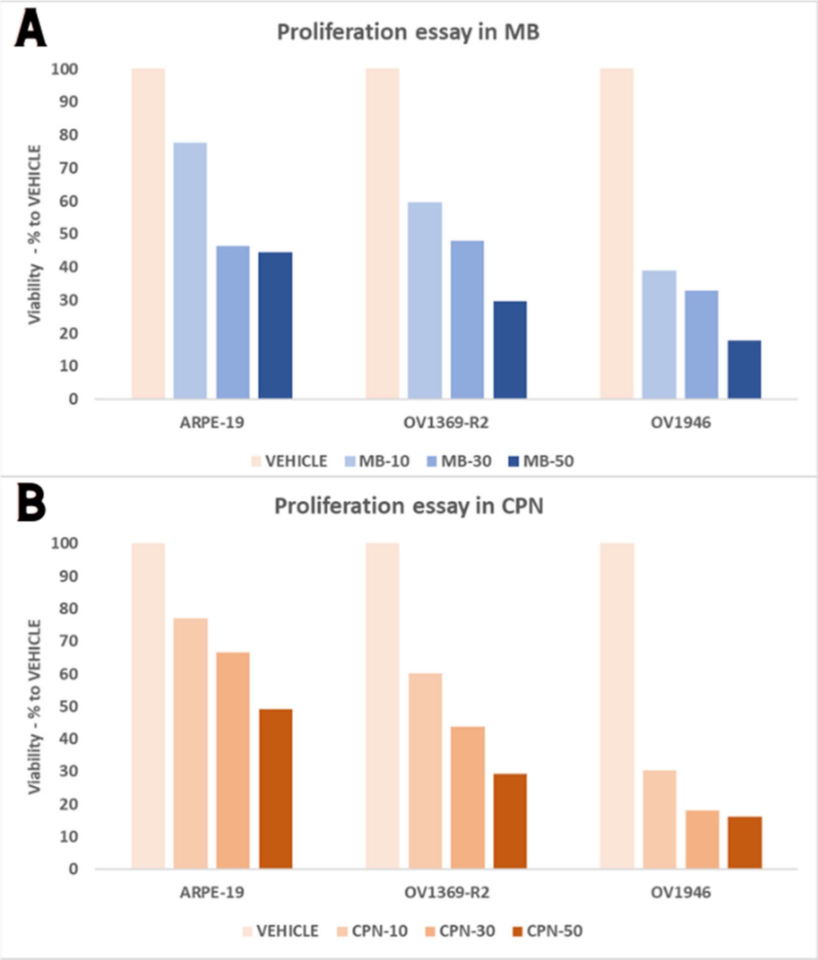

Figure S1. Dose-dependent viability of cancer lines to (A) methylene blue (MB) and (B) carboplatin (CPN) treatments. Ovarian cancer cells are more sensitive to MB and CPN compared to the ARPE-19 control. OV1946 is more sensitive to MB than OV1946-R2, for all three concentrations MB-10, MB-30, and MB-50. The MB IC_{50} for ARPE-19, OV1369-R2 and OV1946 are 24 µM, 25 µM and 7 µM respectively. The CPN IC_{50} for ARPE-19, OV1369-R2 and OV1946 are 48 µM, 18 µM and 5 µM respectively
